# Functionalized Graphene-Based Biosensors for Early Detection of Subclinical Ketosis in Dairy Cows

**DOI:** 10.1021/acsami.4c07715

**Published:** 2024-08-22

**Authors:** Shannon Chick, Matin Ataei Kachouei, Katharine Knowlton, Md. Azahar Ali

**Affiliations:** † School of Animal Sciences, Virginia Tech, Blacksburg, Virginia 24061, United States; ‡ Biological Systems Engineering, Virginia Tech, Blacksburg, Virginia 24061, United States

**Keywords:** Precision livestock farming, Subclinical ketosis, Biosensor, Graphene, Stabilized enzyme

## Abstract

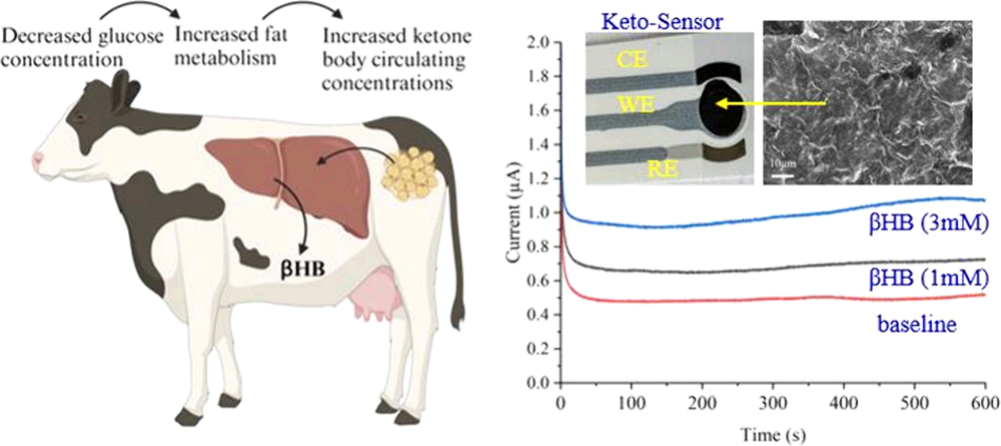

Precision livestock farming utilizing advanced diagnostic tools, including biosensors, can play a key role in the management of livestock operations to improve the productivity, health, and well-being of animals. Detection of ketosis, a metabolic disease that occurs in early lactation dairy cows due to a negative energy balance, is one potential on-farm use of biosensors. Beta-hydroxybutyrate (βHB) is an excellent biomarker for monitoring ketosis in dairy cows because βHB is one of the main ketones produced during this metabolic state. In this report, we developed a low-cost, Keto-sensor (graphene-based sensor) for the detection of βHB concentrations in less than a minute. On this device, graphene nanosheets were layered onto a screen-printed electrode (SPE), and then, a stabilized enzyme (beta-hydroxybutyrate dehydrogenase, NAD^+^, and glycerol) was used to functionalize the graphene surface enabled by EDC–NHS conjugation chemistry. The Keto-sensor offers an analytical sensitivity of 10 nm and a limit of detection (LoD) of 0.24 nm within a detection range of 0.01 μm–3.00 mm. Spike testing indicates that the Keto-sensor can detect βHB in serum samples from bovines with subclinical ketosis. The Keto-sensor developed in this study shows promising results for early detection of subclinical ketosis on farms.

## 1 Introduction

The tools of precision livestock farming offer opportunities to monitor the health status of dairy herds with early detection of disease.^1−3^ On-site sensing technologies may identify early signs of illness minimizing cost and time of treatment. They may be used as actionable decision-making tools for the management of dairy herds.^4^ Subclinical ketosis (SCK)^5,6^ is a metabolic disease in early lactation cows caused by the intense demand for glucose and the mobilization of adipose tissue during a cow’s transition period.^6^ While there is no visual indication of ketosis, elevated ketone concentrations (acetone (Ac), acetoacetate (AcAc), and beta-hydroxybutyrate (βHB)) in all bodily fluids, such as milk and blood, are markers of SCK.^7,8^ If not treated, a ketotic cow will lose her appetite, have decreased production and reproductive performance, and become more susceptible to other diseases such as mastitis, displaced abomasum, and metritis.^9^ It is estimated that the prevalence of SCK is high (∼40–60%) compared to clinical ketosis (∼2–15%)^10,11^ and prevention is valuable because the cost of SCK includes the treatment of the animal, loss of milk production, and delays in conception. Estimates of cost per case vary, ranging from $24 to $1030 per case,^12^ and averaging $165 per case based on stochastic simulation modeling.^13^ Rapid detection of SCK at its earliest stages allows the producer to make better management decisions for their animals and minimize the disease’s economic impact.

The quantification of βHB is known as a gold standard for the diagnosis of SCK in dairy cows.^14^ A dairy cow with concentrations of blood βHB greater than 1.00 mm is considered to have SCK.^15^ Standard thresholds for SCK are 0.10 mm for milk and 1.50 mm for urine.^15^ Traditional diagnostic tools for ketosis include cow-side urine dipstick tests and laboratory-based diagnostic tests. Dipstick tests involve the stimulation of urination onto a stick of paper.^6,16^ The color change on the paper reflects the quantity of ketones present in the cow’s urine. This cow-side test is simple but neither precise nor accurate. Another common tool to detect ketosis involves sending samples of blood, urine, or milk from cows to a laboratory to be analyzed using enzymatic tests based on spectrophotometry.^15,17^ Such assays are accurate^18^ but sample processing and shipping from herds to testing facilities limit their utility for use in making management and treatment decisions. To address these challenges, on-site biosensing tools could be helpful in the management of cow health for their rapid and early diagnosis of SCK.^14^ Accurate, sensitive, and precise biosensing tools would enable quick decisions, on-farm detection, prevention of clinical ketosis, reduce economic losses of the disease, and improve the well-being of cattle. A microfluidic biosensor developed by researchers was able to detect SCK in 1 min with sensitivities in the range of millimolar concentrations (i.e., 0.05 mm).^14,19^ These biosensor tests used quantum dots^19^ and microfluidics with optical coupling.^14^ Additional ketosis tests developed by Ketolac (Biolab, München, Germany),^20^ and Optimum Xceed (Voyvoda and Erdogan)^21^ are commercially available. These hand-held devices were made exclusively for human monitoring; thus, they have limited sensitivities (less than 85%) compared to laboratory tests. One other commercial tool was developed by Novavet^22^ for the detection of ketosis; it has a limited sensitivity of 82% along with significant false-positive results. Thus, there is an unmet need to develop reliable and low-cost ketosis biosensors that allow for the on-farm detection of SCK in dairy cows.

Nanostructures with two-dimensional (2D) geometries are utilized to construct advanced biosensors to detect many biomarkers in biological media.^23−26^ These geometries allow for surface functionalization, whichloads more number of enzymes or antibodies onto the surface of the electrode due to their high surface area,^27,28^ and also enhances the electron transport properties, resulting in rapid detection of the targets. 2D materials, specifically graphene nanosheets, provide abundant functional groups (−COOH) to bind with enzyme molecules (−NH_2_) covalently via amidation reactions.^29,30^ However, the enzymes need to have high stability on the sensor surface for on-farm detection.^31^ Graphene-based biosensors have been used in agriculture to detect microorganisms in food, monitor crop health, and measure biomarkers of disease in livestock, but unstable enzymes limit performance.^32,33^ Thus, enzyme stabilization is a key to success for biosensors,^34−36^ providing advantages such as a) increased sensor-to-sensor reproducibility and sensor shelf life, b) reduced biofouling, c) maintenance of sensor functionalities, and d) reduced tendency of the enzyme to unfold.^34,37^

In this study, we developed a low-cost, and highly sensitive nanoenabled electrochemical biosensor (i.e., Keto-sensor) that detects βHB to identify SCK in dairy cows. This Keto-sensor consists of a carbon-based screen-printed electrode (SPE) which is modified with graphene oxide nanosheets to allow covalent functionalization aided by N-ethyl-N′-(3-(dimethylamino)propyl) carbodiimide (EDC) and N-hydroxy succinimide (NHS) chemistry of enzymes. The sensor utilizes two enzymes, beta-hydroxybutyrate dehydrogenase (βHBD) and β-nicotinamide adenine dinucleotide (NAD^+^), which were stabilized with a glycerol treatment before their immobilization. As expected, enzyme stabilization improved sensor stability and selectivity. We conducted a systematic sensing evaluation of all sensors (S-sensor, G-sensor and Keto-sensor). Upon calibration, the spiking analysis with complex biological matrices, including bovine serum and discarded milk, showed good validation. Spiking analysis involved adding a known concentration of the analyte into a sample that is close to or identical to real samples. Results show a promising alternative diagnostic tool that detects SCK in dairy cows.

## 2 Materials and Methods

### Chemicals and Reagents

Graphene oxide (ACS Material, CA, USA) was used as a base layer for enzyme functionalization onto the working electrode (WE) of the G-sensor and Keto-sensor. Phosphate buffered saline (PBS) solution was used to prepare stock solutions of NHS, EDC, βHBD, NAD^+^, and βHB, as well as the buffer solution to establish a sensor baseline. Fetal bovine serum (FBS, Sigma-Aldrich, MO, USA) was used for spiked sample testing as serum could be a potential matrix to measure βHB. βHB was used to make standard titration solutions (0.01 μm–3.00 mm) to calibrate the Keto-sensor. βHBD, a specific enzyme to βHB, was immobilized onto the graphene oxide surface via EDC-NHS chemistry. Within the enzyme solution were NAD^+^ and glycerol. NAD^+^ takes part in the oxidation–reduction reaction that occurs when βHBD catalyzes βHB and glycerol is a stabilizing agent for βHBD. All chemicals such as βHBD, βHB, NAD^+^, EDC, NHS, and glycerol were purchased from Sigma-Aldrich, MO, USA. Further explanation of how stock solutions were prepared can be found in the Supporting Information (see Section 1).

### Instrumentation

**Figure 1:**
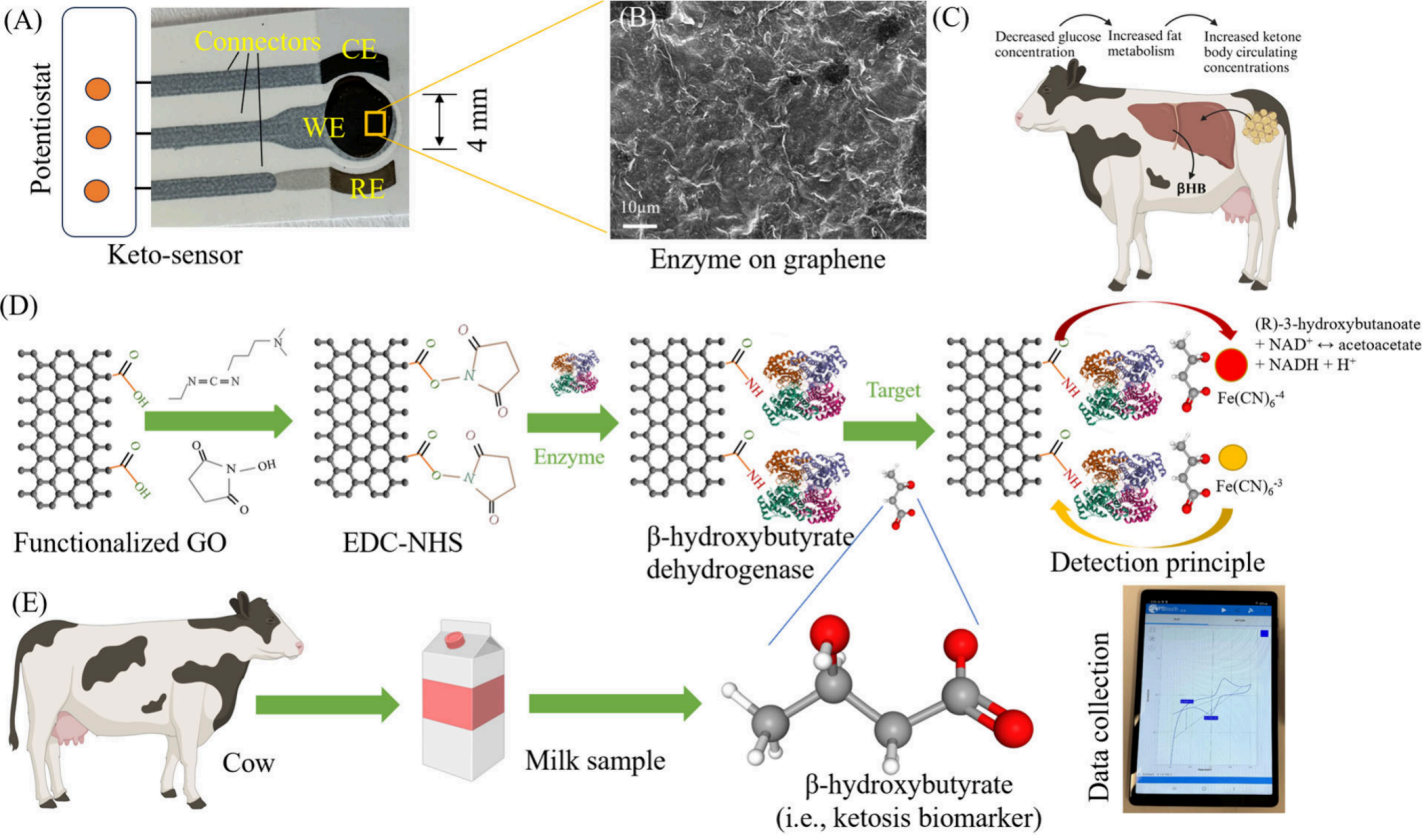
Schematic of a Keto-sensor to measure βHB in dairy cows. (A) Keto-sensor setup displaying the connectors, working electrode (WE), counter electrode (CE), and reference electrode (RE). (B) SEM image of the enzyme that was immobilized onto the graphene/WE. (C) Visual representation of how decreased glucose levels lead to elevated fat metabolism and increased circulating βHB concentrations in dairy cattle. (D) Schematic showing the creation of the WE for the Keto-sensor. Functionalized graphene coats the carbon substrate of the screen-printed electrode (SPE). Once the graphene was dried onto the surface of the substrate, then an EDC–NHS solution was added and allowed to sit in a humid chamber. After 4 h, the excess EDC–NHS was washed from the surface and the βHBD enzyme solution was added and allowed to sit in the humid chamber for up to 12 h. Both βHBD and NAD^+^ were added as a bienzyme during the preparation of the enzyme solution. When testing the sensor, the βHB in the sample reacted with the βHBD. (E) Visual representation of a dairy cow producing milk that can be tested for βHB as a biomarker of SCK.

Carbon based SPEs (Palm Sens, Netherlands) were used to build sensors. These SPEs used a graphite WE with a 3 mm diameter, a carbon counter electrode (CE), and a silver reference electrode (RE). The SPEs had small wrinkles on the surface of the WE, which created a greater surface area for sensing analytes. The use of commercial electrodes avoided clean-room fabrication, reduced the device cost, and ensured the reproducibility of the sensor. These sensors can be easily connected to a potentiostat that can send data readouts via Bluetooth. Sensors were inserted into a commercial potentiostat (EmStat Blue, Palm Sens, Netherlands) to conduct experiments (Figure S1, Supporting Information). The Bluetooth-enabled potentiostat was connected to a computer or tablet to collect data (PSTrace, Palm Sens, Netherlands) and data were exported to Origin. Inc. (OriginLab, MA) to create graphs. BioRender was used to create the schematics in Figure 1.

### Device Fabrication

The Keto-sensor in this study has CE, WE, and RE (Figure 1), and the electrodes were screen-printed onto a paper substrate. The WE for this Keto-sensor was modified. A photo of a Keto-sensor is shown in Figure 1A. A solution containing highly dispersed 2D graphene oxide nanosheets was pipetted onto the WE and dried for 1 h at 80 °C. This procedure was done twice for a uniform surface of graphene oxide. During this process, graphene oxide nanosheets were layered onto the carbon SPE due to the noncovalent π–π interactions of graphene and carbon.^38^ Next, EDC and NHS solutions were applied at a one-to-one ratio to the WE and the sensor was placed in a humid chamber for 4 h.^39^ After 4 h, the electrode was washed with commercially available PBS (Gibco, MA) solution, and the sensor was functionalized with enzymes. This EDC–NHS treatment^40^ activated the abundant −COOH groups on the graphene oxide modified Keto-sensor to bind with proteins.

For enzyme functionalization, an enzyme solution was prepared consisting of NAD^+^ and βHBD mixed at a 1:1 ratio. To stabilize the enzyme, glycerol was mixed into the final enzyme solution as 5% of the final volume. Twenty μL of this solution was spread uniformly on the surface of the graphene oxide electrode. In this EDC–NHS chemistry, −COOH groups on the graphene surface bind to −NH_2_ of the enzyme and form a C–N covalent bond via an amidation reaction.^29^ The EDC reacted with the graphene −COOH groups and formed o-acylisourea, which can react with the NHS resulting in NHS esters. These NHS esters can react with amines of the enzyme to form a C–N covalent bond.^29^ These functionalization steps are visualized in Figure 1D. The sensor was placed in a humid chamber for at least 4 h, but for no longer than 12 h. After incubation, the electrode was washed again with PBS, then the sensor was placed in a 4 °C refrigerator until use.

The potentiostat attaches to the three connectors that lead to the CE, WE, and RE as seen in Figure 1A and Figure S1 (Supporting Information). On the WE are layers of graphene oxide with enzyme, as represented by the scanning electron microscopy (SEM) images in Figure 1B. A cow’s blood glucose concentrations decrease after calving because of the negative energy balance caused by the sudden onset of lactation and the limited food consumption typically observed. This negative energy balance triggers metabolism of body fat to provide energy, and ketone bodies like βHB are produced in the process. Elevated blood ketone concentrations further impair feed intake, and this cycle often leads to SCK. This process, of a dairy cow developing ketosis after calving, is outlined in Figure 1C.^41^ Graphene oxide was added to the screen-printed WE, EDC–NHS coupling with the graphene was done as described above, the enzyme solution was added to the sensor, and finally, sensing was performed with this functionalized Keto-sensor. βHBD catalyzes the βHB to acetoacetate. The role of NAD^+^ in this reaction is to act as a cofactor for the reaction creating βHB.^42^ The glycerol in the enzyme solution ensures stability over time.^43^ Milk, and blood samples can be collected from the cows and used to monitor βHB as outlined in Figure 1E. In addition to the Keto-sensor, two more sensors were chosen as controls in this study. These control sensors were the S-sensor (SPE-based sensor) and the G-sensor (graphene oxide-modified screen-printed sensor); these sensors do not contain specific enzymes, i.e., βHBD. The carbon-based SPE was composed solely of carbon on the WE (see Supporting Information, Figure S1B). In contrast the G-sensor incorporated layers of graphene oxide onto the WE. The Keto-sensor, akin to the G-sensor, included graphene oxide layers and additionally immobilized the enzyme solution onto these layers. A schematic diagram showing the differences between the WEs of each sensor is included in the Supporting Information (Figure S2). In the diagram, the enzyme solution is depicted by beta-hydroxybutyrate dehydrogenase, which is pivotal in catalyzing the ketone body βHB.

## 3 Results and Discussion

### Surface Morphologies

**Figure 2:**
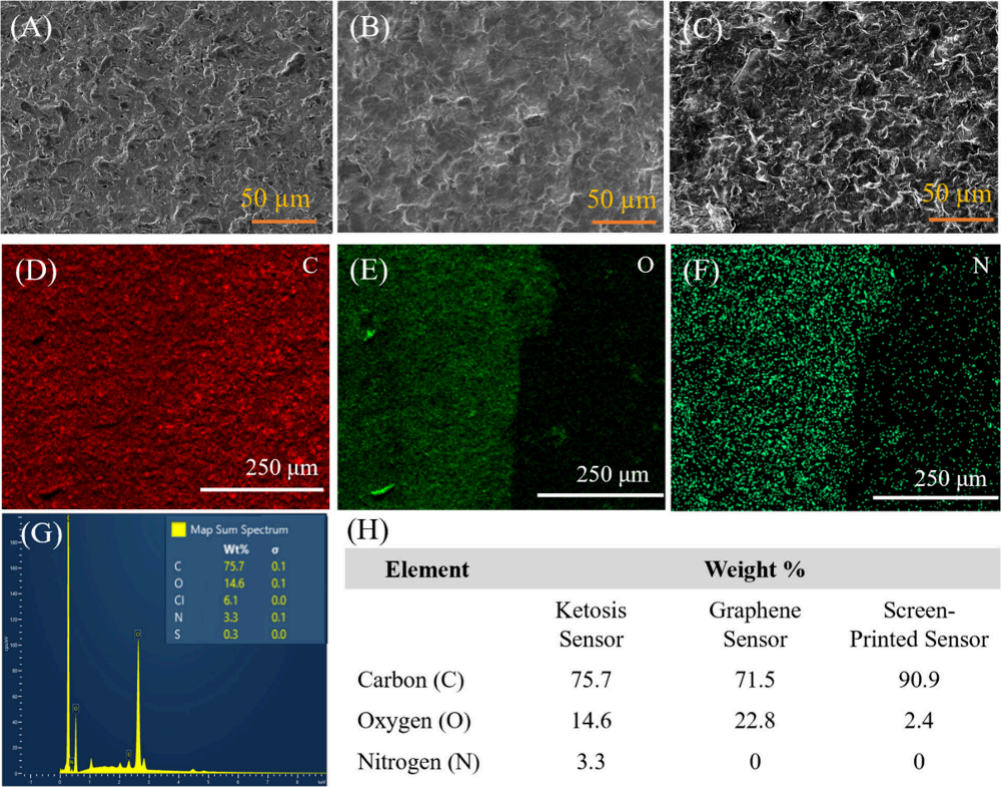
Investigations of the surface morphologies of the sensors. SEM images for screen-printed sensor without modification (A), graphene sensor without enzyme (B), and graphene nanosheets along with enzyme or Keto-sensor (C). (D–F) EDS mapping of the WE of the Keto-sensor showing carbon, oxygen, and nitrogen distribution. (G) Graph showing weight distribution of different elements found on the WE of the Keto-sensor. (H) Table showing the weight distribution of the different elements found on the WE of the ketosis sensor.

To investigate the surface morphologies of the sensors, we conducted SEM imaging along with energy-dispersive X-ray spectroscopy (EDS) using the JEOL IT500 SEM and Oxford Instrument AZtechOne Detector. The SEM imaging was conducted for the screen-printed sensor without modification (S-sensor) (Figure 2A), the graphene oxide sensor without enzyme (G-sensor) (Figure 2B), and the graphene oxide nanosheets along with enzyme (Keto-sensor), (Figure 2C) at 500X. The same sensors used as SEM samples were coated with a thin layer of iridium for EDS analysis. The SPE showed the bare bulky carbon structure that is packed together with a non-uniform surface (Figure 2A). This morphology was changed with nanosheets of graphene oxide layers (Figure 2B). These nanosheets were seen to be connected and formed a porous, thick layer. This porous layer was expected due to the π–π interactions among the nanosheets of carbon. Some nanosheets formed wrinkles due to stacking and folding. This nanoenabled sensor surface not only increased the area of surface reactions but also enhanced the loading of enzymes. The morphology was further changed when enzymes were immobilized via EDC–NHS chemistry (Figure 2C). The enzyme layer on the graphene oxide, however, was unclear to observe.

Figure 2D, E, and F displays the mapping results from the EDS of the Keto-sensor to evaluate individual elements. The EDS spectrum of the Keto-sensor indicates the presence of respective elements (Figure 2G). The surface was completely covered in carbon (C ∼ 90.9%) as the SPE used a carbon base (Figure 2D). Oxygen (O) was dispersed across the surface of the SPE as the WE were modified with graphene oxide nanosheets **(**Figure 2D**)**. The distribution of nitrogen (N) on the WE correlate to where the enzyme was attached to the graphene oxide layer (Figure 2F). The presence of N indicated enzyme immobilization on the sensor surface. The addition of graphene oxide nanosheets to both the Keto-sensor and G-sensor was likely the cause of the increase in the O weight percentage (wt %) of the WE as compared to the S-sensor seen in Figure 2H. N was present on the WE of the Keto-sensor, but not on the WEs of the S-sensor and G-sensor due to the addition of the enzyme, βHBD, to the Keto-sensor (Figure 2H). A table comparing the three different sensors is shown in Figure 2H.

### Electrochemical Sensing of Ketosis and Characterization

**Figure 3:**
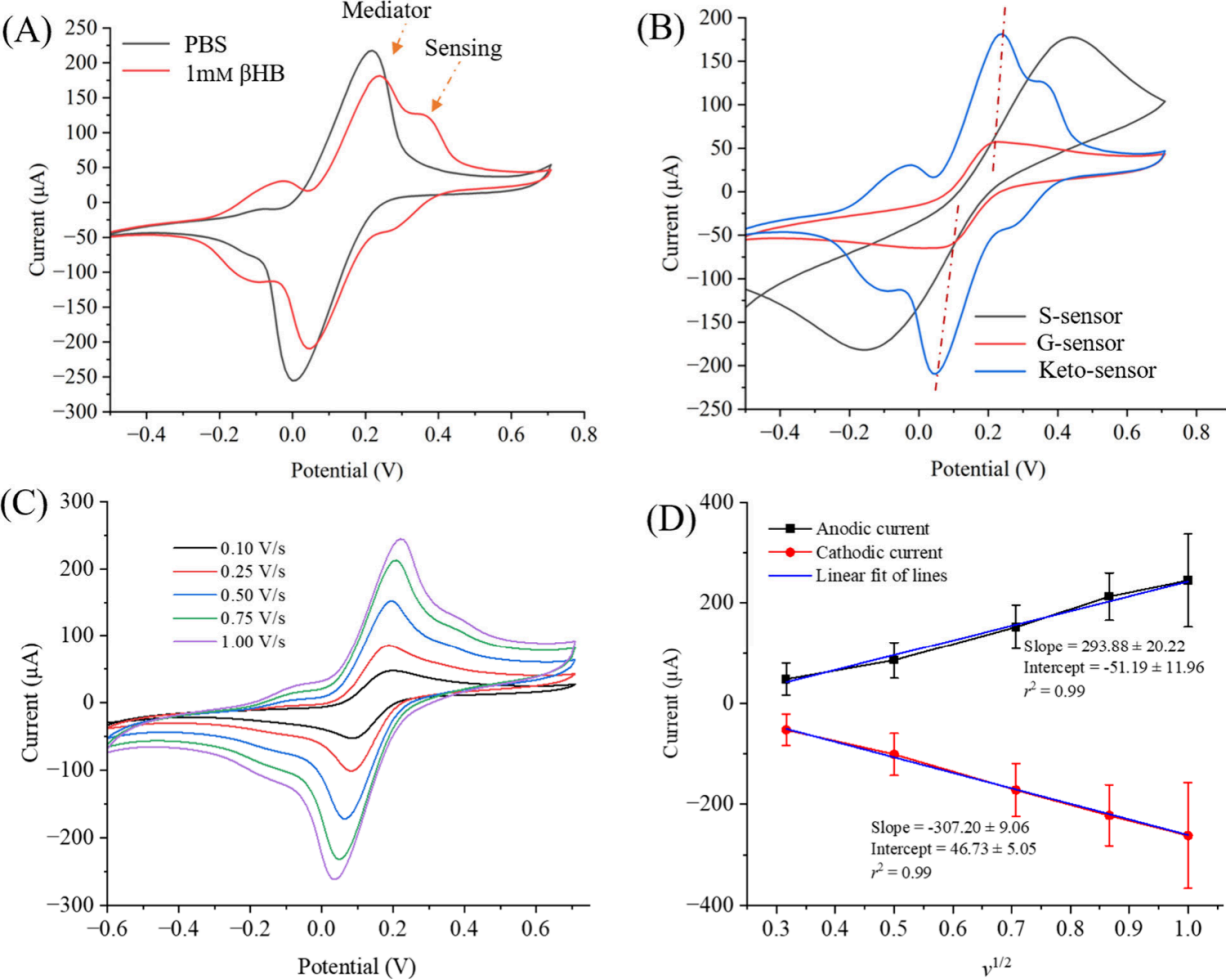
Electrochemical characterization of the sensors. (A) Cyclic voltammetry (CV) of the Keto-sensor with and without βHB (1 mm) concentration. For the CV test, the PBS solution contains a 5 mm concentration of a [Fe(CN)_6_]^3–/4–^ redox mediator. (B) CV comparison for the S-sensor, G-sensor, and Keto-sensor in the presence of βHB (1 mm) concentration. (C) Scan rate study for Keto-sensor wherein the scan rate was varied from 0.10 to 1.0 V/s. As the scan rate increases, the peak currents increase. (D) The calibration plot of scan rate studies compares the peak current to the square root of the scan rates.

**Figure 4:**
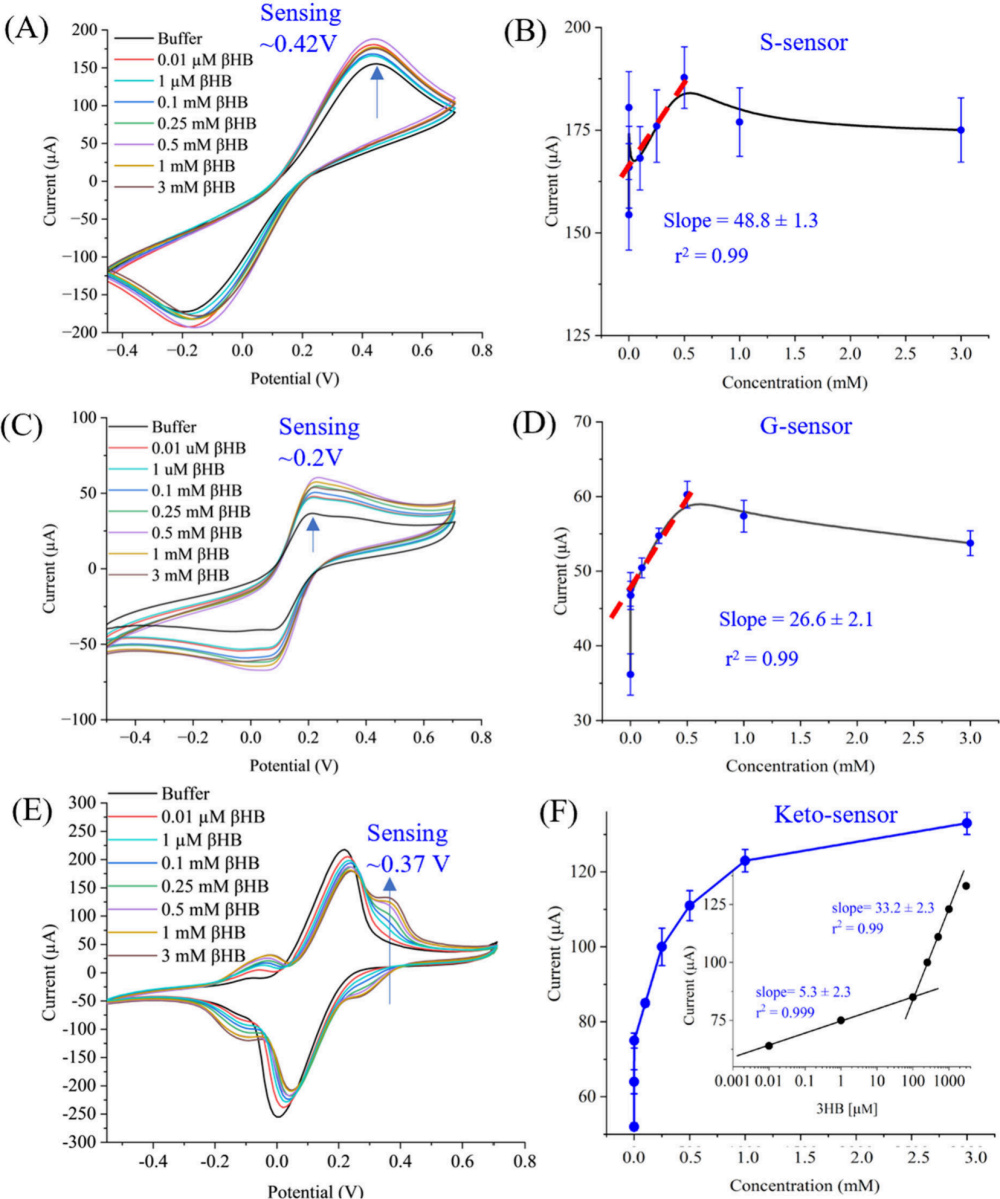
Sensing performance of the sensor. All dilution studies were conducted at room temperature and used the same βHB serial dilutions, and buffer solution (0.01 μm, 1.00 μm, 0.10 mm, 0.25 mm, 0.50 mm, 1.00 mm, and 3.00 mm) for all the sensors. (A, B), (C, D), and (E, F) present the sensing performance of the S-sensor, G-sensor, and Keto-sensor, respectively. A, C, and E show the CV responses for the S-sensor, G-sensor, and Keto-sensor, respectively, with increasing concentrations of βHB in buffer solution. B, D, and F are the sensor calibration plots for S-sensor, G-sensor, and Keto-sensor, respectively. In all three sensors, the increase in βHB concentration led to an increase in peak current values. Data shown in B, D, and F were produced by the three repeated measurements (*n* = 3) of biologically independent concentration of targets. Error bars are calculated by taking the standard deviations of the three repeated measurements.

Electrochemical studies were conducted to investigate the redox properties of the sensors: the Keto-sensor, S-sensor, and G-sensor. The cyclic voltammetry (CV) measurements of all sensors were conducted using a PBS solution containing an equimolar concentration (5 mm) of a ferro/ferricyanide redox mediator. The cyclic voltammograms of the Keto-sensor in the absence and presence of 1.00 mm βHB as the target analyte demonstrate the ability of the Keto-sensor to detect βHB (Figure 3A). A pair of oxidation and reduction peaks in both graphs was related to the redox reaction of the mediator on both sensors. However, in the presence of βHB (1.00 mm), the Keto-sensor showed a decrease in the redox peaks and a significant shift toward greater oxidative potential. Additionally, a dominant oxidation peak appeared at 0.37 V (vs Ag/AgCl) in the presence of βHB. This peak is considered the sensing signal for the measurements of βHB concentration. As the Keto-sensor contains enzymes that catalyze the βHB to acetoacetate and produce electrons at an oxidation potential of 0.37 V, this confirms that the Keto-sensor selectively detected the presence of βHB in buffer solutions. Another peak was observed at nearly 0.00 V due to the unreacted EDC-NHS on the surface of the graphene oxide that was used in the sensor construction. The CV tests showed a clear difference between the S-sensor, G-sensor, and Keto-sensor in the presence of 1.00 mm βHB (Figure 3B). The S-sensor was a bare SPE sensor without any modification. This sensor showed strong oxidation and reduction peaks in the presence of βHB (1 mm) in the buffer solution which was attributed to the redox peaks of ferro/ferricyanide mediators. The oxidative peak potential and current were at ∼0.42 V and 150 μA. When the electrode surface was modified with graphene oxide nanosheets (G-sensor), this mediator oxidation peak potential decreased to 0.30 V and the current decreased (50 μA) significantly. This lower current was due to the available functional groups at the graphene surface that block the electron transfer from the mediator to the electrode. The differing performance of the G-sensor and S-sensor in detection of βHB concentration is presented in Figure 4. Though the current was lowered with the G-sensor, this configuration was used to add the stabilized enzyme to the WE due to its lower sensing potential and ability to enhance the selectivity of the sensor. In more oxidative potentials, the electro-oxidation of interfering molecules in the complex matrix of the real sample can affect the detection of βHB. The Keto-sensor had notable multiple oxidation and reduction peaks in comparison with the S-sensor and G-sensor (Figure 3B**)**. The addition of the enzyme on the Keto-sensor created these additional peaks, indicating that the enzyme was reacting with the analyte causing the exchange of electrons, resulting in high selectivity. Ultimately, the Keto-sensor boosts the sensing signal more than three times compared to the G-sensor that can diminish the redox reactions due to insulative effects. The promoted electron exchange properties of βHBD were due to the enzymatic reactions during the detection of βHB concentration. Unlike the Keto-sensor, the G-sensor and S-sensor did not show peaks other than the mediator redox peaks in their respective CV graphs (Figure 3B) during sensing of βHB.

A scan rate study was performed using the Keto-sensor. For this study, a range of potential from −0.70 to 0.70 V (vs Ag/AgCl) was applied at different potential scan rates on multiple sensors (n = 5). Each scan rate was tested three times on each sensor. The results of scan rate studies that were conducted using the Keto-sensor and tested scan rates of 0.10, 0.25, 0.50, 0.75, and 1.00 V/s are displayed in Figure 3C and D. With the increase in scan rate, the oxidation peaks during the cyclic voltammetry increased, indicating the surface-controlled process of the Keto-sensor. With a change in scan rate, there was a shift in the oxidation peak current to the right and a shift in the reduction peak current to the left with increasing scan rate that increases the Δ*E*_p_. The peak currents were linearly proportional to the square root of the scan rate, rather than the scan rate, indicating that the surface reactions were diffusion controlled (Figure 3D) process. The sensing performance of all three sensors was investigated using electrochemical characterizations. Though the Keto-sensor provided comparable sensing with the S-sensor, it is expected that the Keto-sensor can provide more selectivity due to the added enzyme layer that can generate electrons directly from the electro-oxidation of βHB species. The lower sensing potential of the Keto-sensor is another advantage compared to the S-sensor that can boost selectivity.

### Ketosis (βHB) Sensing

Enzymatic and nonenzymatic sensing performances were investigated for the S-, G- and Keto-sensor. In the nonenzymatic sensing modality,^42^ the S- and G-sensors performed electrocatalytic reactions without any enzymes, while the enzymatic Keto-sensor^44^ performed enzymatic oxidation resulting in electron generation in the presence of βHB. Carbon^45^ and graphene^46^ can both act as electrocatalysts. These are useful materials to construct nonenzymatic sensors but the selectivity in their performance is limited in comparison with enzymatic sensors. This causes challenges in complex sample matrices like milk or serum. A CV test was used to calibrate all the sensors at a scan rate of 1.00 V/s. Several dose-dependent standard solutions of βHB (0.01 μm, 1.00 μm, 0.10 mm, 0.25 mm, 0.50 mm, 1.00 mm, and 3.00 mm in buffer solutions) were prepared. This covers the physiological range of βHB (SCK∼ blood 1.20–2.90 mm and clinical ketosis ∼ ≥ 3.00 mm) in dairy cows.^47^ Testing samples were prepared using a PBS (50 mM, and pH∼ 7.4) solution containing an equimolar concentration (5 mm) of ferro/ferricyanide. This buffer without βHB was used to establish the sensor’s baselines. Sensing measurements for all sensors are shown in Figure 4. For each concentration, three repeated measurements (n = 3) were taken with biologically independent solutions. Error bars were calculated by calculating the standard deviation of three measurements.

βHB sensing results for the S-sensor were collected using dose-dependent concentrations (Figure 4A, B**)** using CV measurements (Figure 4A). The levels measured for all sensors are within the physiological range of both subclinical and clinical ketosis.^48^ Subclinical levels are around 1.00 mM while clinical ketosis levels are 3.00 mM or greater in bodily fluids. The sensor was also tested versus concentrations below the subclinical level, which opens the opportunity for continuous testing of individual animals to monitor ketone levels before they reach subclinical status. Farmers can then try to mitigate the negative energy balance that can cause ketosis before the animals reach that metabolic state. Initially, the S-sensor was exposed to a buffer solution to set the sensor baseline. Clear oxidation and reduction peaks were observed due to the presence of a ferro/ferricyanide mediator. The oxidation current was found to be at 155 μA. Next, a minimum concentration of βHB (0.01 μm) was introduced to the S-sensor. The peak current was enhanced significantly compared to the sensor’s baseline. This is due to the nonenzymatic electrocatalysis oxidation of βHB molecules on the carbon surface. Then, the S-sensor was washed with buffer solution before the next solution with a higher concentration was introduced. The βHB concentration was increased from 0.10 μm to 3.00 mm and signal increases (peak current) were observed directly proportional to the increased concentrations of βHB (Figure 4B). However, the S-sensor signal became saturated after 0.5 mm concentration of βHB. This is one of the major limitations of the S-sensor and is overcome by introducing graphene oxide layers along with a stabilized enzyme on the sensor surface. From the sensor calibration, the slope value was estimated as ∼48.8 ± 1.3 μA.

Similarly, sensing measurements were conducted for the G-sensor in the presence of all standard target concentrations of βHB (Figure 4C, D. On adding a graphene oxide layer, the oxidation peak potential was drastically reduced to 0.20 V. Furthermore, the peak current of the baseline’s G-sensor was also reduced to 33 μA. The lesser baseline signal was due to the presence of functional groups on the graphene oxide sheets that impede the electro-oxidation of βHB on its surface.^49^ Then, a low concentration of βHB (0.01 μm) was introduced, and the peak current of the G-sensor was increased notably. Further, the concentration of βHB was increased and the peak current was increased at a potential of 0.20 V. The peak current was found to increase until a 0.50 mm concentration of βHB, after which the G-sensor showed saturated results like those observed with the S-sensor. For this sensor, the slope of the calibration was estimated as ∼26.6 ± 2.6 μA (Figure 4D). The limit of detections (LoDs) for both the S-sensor and G-sensor were estimated as 857.02 nm, and 545.56 nm, respectively. The detailed calculation of LoD is demonstrated in the Supporting Information (Section SI). The analytical sensitivities of the S- and G-sensors were 0.01 μm, but these sensors were not sensitive beyond 0.50 mm concentration of βHB.

After these experiments, sensing characterization of the Keto-sensor was investigated using the same range of βHB concentrations (Figure 4E, F). At a specific potential (0.37 V), the peak current increased while the concentration of βHB concentration increased. Due to the enzyme layer in the Keto-sensor, a distinct peak sensing appeared at potential 0.37 V which was separate from the main redox peak in the CV graph attributed to the mediator redox reaction (Figure 4E). This feature of the Keto-sensor helps to detect a wider range of βHB concentrations. In the Keto-sensor, the enzymatic reaction that takes place on the WE involve βHB and NAD^+^ in an oxidation–reduction reaction. The βHB is oxidized to make acetoacetate, providing an electron to reduce NAD^+^ to NADH.^42^ In the reverse of this reaction, NADH provides the electron to reduce the acetoacetate back into βHB.^50−52^ The reaction can be written as (R)-3-hydroxybutanoate + NAD^+^ ↔ acetoacetate + NADH + H^+^.^50^ This enzymatic reaction is responsible for increasing the electrochemical current during sensing of βHB. Additionally, on all sensors, the reaction K_4_Fe(CN)_6_ ↔ K_3_Fe(CN)_6_ takes place on the WE. The Keto-sensor in this study showed two linear ranges of detection. One was the detection of low concentration from 0.01 μm to 100 μm with a slope value of 5.3 ± 2.3 μA and the other was from 100 μm to 3.00 mm with a slope value of 33.3 ± 2.3 μA. The Keto-sensor shows a logarithmic relationship between the sensor current (i) and the concentration of βHB. This indicates that as the concentration of βHB increases, the sensor current increases in a logarithmic manner. Further, the small changes in concentration at lower levels result in larger changes in sensor current, while at higher concentrations, larger changes in concentration are needed to produce the same increase in current. On the other hand, the S-sensor and G-sensor exhibit a linear relationship between sensor current and concentration of the target analyte. This indicates that the sensor current increases linearly with the concentration of the analyte. The logarithmic response of the Keto-sensor to βHB concentration is due to the nature of the underlying biochemical reaction or mechanism that occurs within the sensor’s design. This is primarily caused by the presence of enzymes on the sensor’s surface. At lower concentrations of βHB, the enzyme active sites are not fully occupied, leading to a rapid increase in the reaction rate and thus the sensor current increases as βHB concentration increases logarithmically. However, as βHB concentration increases further, the enzyme active sites become saturated, causing the reaction rate, and hence the sensor current, to increase more slowly or plateau. We believe that the transition in slope from low to high concentrations was primarily attributable to the enzyme activity and the availability of binding sites on the enzyme.^53^

**Figure 5:**
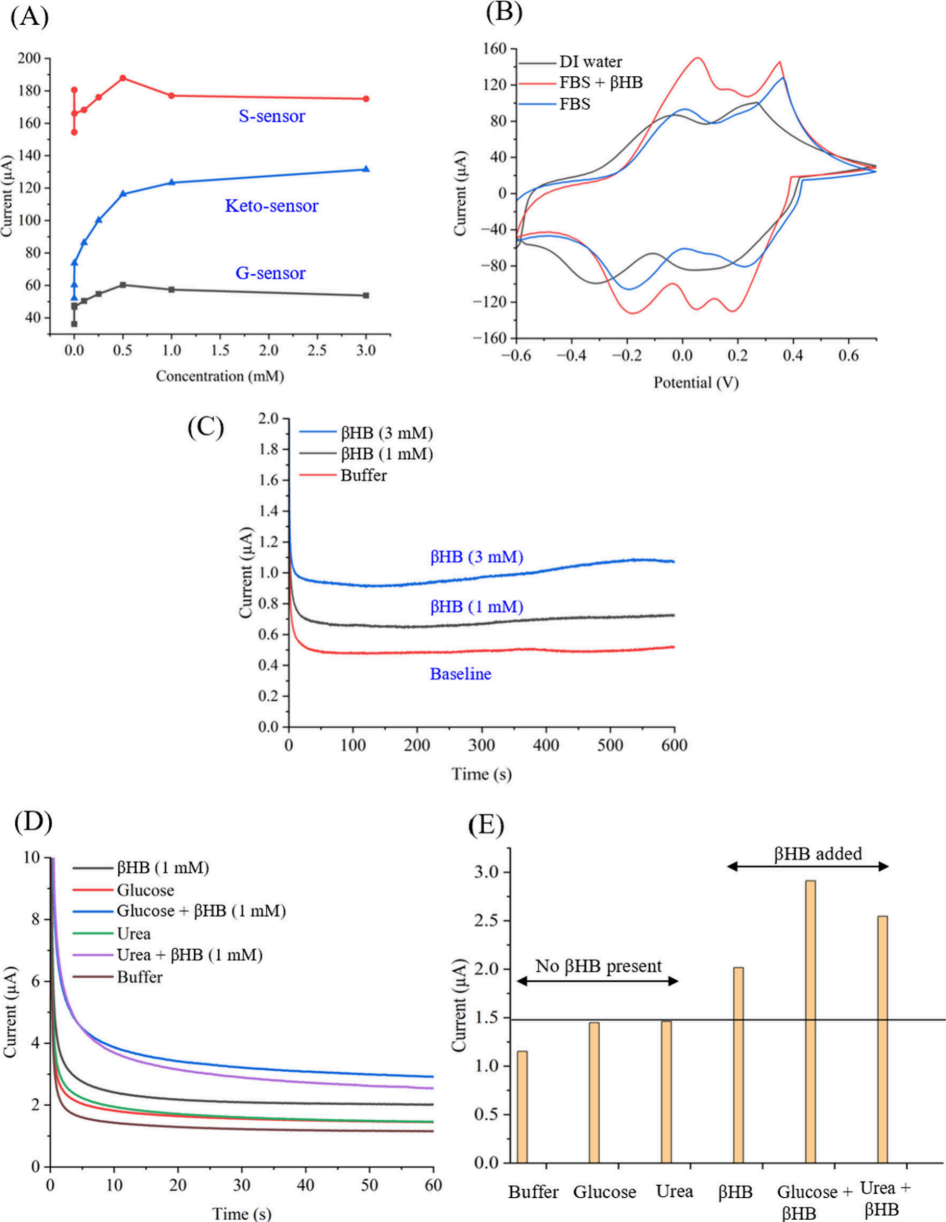
Studies with real samples, continuous, and selectivity measurements. (A) A comparison plot showing the calibration plots for S-sensor, G-sensor, and Keto-sensor. (B) Cyclic voltammetry graph of Keto-sensor testing deionized water, fetal bovine serum, and fetal bovine serum spiked with 3 mm of βHB. The addition of βHB to the serum increases the peak values recorded during CV. (C) Chronoamperometry graph showing long-term and continuous sensing of 3 mm βHB, 1 mm βHB, and buffer solutions. For this study, we continuously ran 10 min of measurements. (D) Chronoamperometry graph for selectivity study on the Keto-sensor. Different solutions of urea and glucose excluding and including βHB were added to the sensor and chronoamperometry was performed for 1 min (60 s) with each solution. Between each addition, the sensor was washed with PBS. (E) Bar graph based off the selectivity study. Solutions with βHB present show a higher current versus those without βHB present.

This large increase in slope reflects the increase in concentration of βHB from the micromolar range to the millimolar range. The LoD of this Keto-sensor was estimated as 0.24 nm (see the calculation in Supporting Information, Section 2). This Keto-sensor has a high detection range compared to the other S-sensor and G-sensor which is due to adding an enzyme layer on the sensor surface (Figure 5A). With the curves overlaid it was clear that the Keto-sensor has the best differentiation between different concentrations of βHB. With the S-sensor and G-sensor, the higher concentrations of βHB were harder to distinguish as sensors become saturated.

FBS was measured with and without βHB added (Figure 5B**)**. This spiked sample testing involved adding concentrations of βHB into FBS to assess the effectiveness of the Keto-sensor in a more complex matrix. The Keto-sensor showed the same shape CV graphs containing two dominant peaks with and without βHB when compared to measurements of FBS-only and DI water samples. The FBS may contain βHB prior to spiking, as ketone bodies can be found in all bodily fluids, thus the sensor showed a peak current at ∼0.40 V. When the FBS solution was spiked with a higher concentration (3.00 mm) of βHB, the peak current increased at the same potential, indicating the Keto-sensor can selectively detect βHB in the complex matrix of bovine serum.

For long-term and continuous measurement testing, chronoamperometry was conducted in buffer and different standard concentrations of βHB (Figure 5C). The Keto-sensor was first set to a baseline without any βHB. Then, 50 μL of solution was pipetted onto the sensor and the current was measured continuously for 10 min. The Keto-sensor was tested with βHB concentrations of 1.00 mm and 3.00 mm and compared to the buffer solution. A potential of 0.37 V was applied to each sensor for the 10 min of measurement. With this experiment, the data shows that the Keto-sensor can detect concentrations of βHB that would be considered normal as well as those indicating SCK over extended periods of time. These results show promise for the possibility of continuous monitoring with these sensors. Farmers could use this device to monitor animal health with clear differentiation between healthy cows and those with SCK. This monitoring would allow management decisions to both prevent and treat the disease.

Table I compares the Keto-sensor created during this study with sensors developed by others for detecting βHB. Many of the devices made to measure βHB focus on use with human patients, however, this device and one other^54^ were made with the intended goal of on-farm use. Many of these devices used amperometry for measuring,^54−56^ while this device and one other^57^ used cyclic voltammetry measurements. The limit of detection for the Keto-sensor was 0.24 nM, while the limit of detection for the G-sensor and S-sensor were 545.56 nm and 857.02 nm, respectively. The Keto-sensor has a much lower limit of detection than all other sensors (Table I). The wide range of detection (0.01 μm to 0.10 mm and 0.25 to 3.00 mm) allows the Keto-sensor to differentiate between healthy dairy cows and those with SCK and clinical ketosis. This capability shows the advantages of the Keto-sensor using graphene oxide nanosheets with a stabilized enzyme on the WE. Further, long-term and continuous measurement of βHB is another important feature of the Keto-sensor compared to others reported in the literature.

**Table I:** Comparison of Sensor Performance with Other Report Literature

Electrode materials	Biochemical reactions	Sensing modalities	Linear Range and LOD	Test samples	Reference
Functionalized graphene	βHB + NAD^+^ (βHBD) → NADH. Detect. NADH	CV +370 mV	0.00001–0.1 mm and 0.25–3.0 mm /0.24 nm	Spiked bovine serum	This work
Graphene	βHB + NAD^+^ (βHBD) → NADH. Detect. NADH with [Ru(bpy)_3_]^2+^	Amperometry +60 mV vs Ag/AgCl	0.2–2.0 mM/–	Bovine serum	Veerapandian et al., 2016
Iridium functionalized carbon	βHB + NAD^+^ (βHBD) → NADH Detect. NADH	Amperometry +200 mV vs Ag/AgCl	0–10 mm/–	Bovine serum	Fang et al., 2008
Reduced graphene	βHB + NAD^+^ (βHBD) → NADH Detect. NADH with THI	Amperometry 0 mV vs Ag	0.003–0.4 mm/0.001 mm	Spiked human serum	Martínez-García et al., 2017
Carbon nanotube	βHB + NAD^+^ (βHBD) → NADH. Detect. NADH	CV −150 mV vs Ag/AgCl	0.01–0.1 mm /0.009 mm	Human serum	Khorsand et al., 2013

We have conducted a thorough comparison and discussion of our findings with established methods, such as liquid chromatography–mass spectrometry (LC-MS),^58^ commonly used for detecting βHB. Recent studies have reported a limit of detection of 3 μM for βHB using LC-MS, while another study utilizing gas chromatography–mass spectrometry (GC-MS)^59^ indicated a limit of detection of 19.2 μM for the same analyte. In contrast, our sensor demonstrates an exceptional lower limit of detection of 0.24 nM for βHB sensing. This significant sensitivity surpasses that of traditional chromatography-based methods. Importantly, while these sophisticated analytical tools offer high accuracy, they are impractical for on-site testing of ketosis due to the need for multiple steps of sample processing, shipping, and their associated high costs. Our sensor presents a practical alternative, enabling rapid, sensitive, and cost-effective detection of βHB directly at the point of need. This positions our technology as a promising tool for timely and accessible ketosis monitoring.

### Selectivity Studies

For the selectivity test, potential coexisting target molecules present in serum samples from dairy cows were applied to the sensors (Figure 5D, E). These molecules can undergo a nonenzymatic electro-oxidation reaction and interfere with the βHB detection. Glucose (2.00 mg/mL; Sigma-Aldrich, MO) and urea (2.50 mg/mL; Fisher Chemical, MA) solutions, with or without 1.00 mm added βHB, were used to test for selectivity. First, the Keto-sensor was set to the baseline. On exposure to glucose and urea, the Keto-sensor did not show a significant change, as expected. The Keto-sensor provided a sensing signal when βHB was added. When βHB was added to glucose and urea solutions, the Keto-sensor showed a slight change in response current. However, the relative standard deviation (RSD) was estimated as ±9.1%. Such low RSD indicated that the Keto-sensor was selective, able to detect βHB even in the presence of other compounds. Figure 5D demonstrates the chronoamperometric graphs and Figure 5E is a bar graph made from Figure 5D to show the difference in current measured from each solution. The stabilized enzyme was responsible for such selectivity as this enzyme can only have biochemical reactions with βHB during detection. An additional study testing selectivity in the presence of uric acid was conducted and these results are presented in the Supporting Information (Figure S3). A uric acid solution of 13.00 mg/L concentration was used for this study. The uric acid solution containing βHB (1.50 mm) exhibited a notable increase in current compared to solutions without βHB, demonstrating high selectivity in the presence of uric acid.

### Real Milk Sample Studies

**Figure 6:**
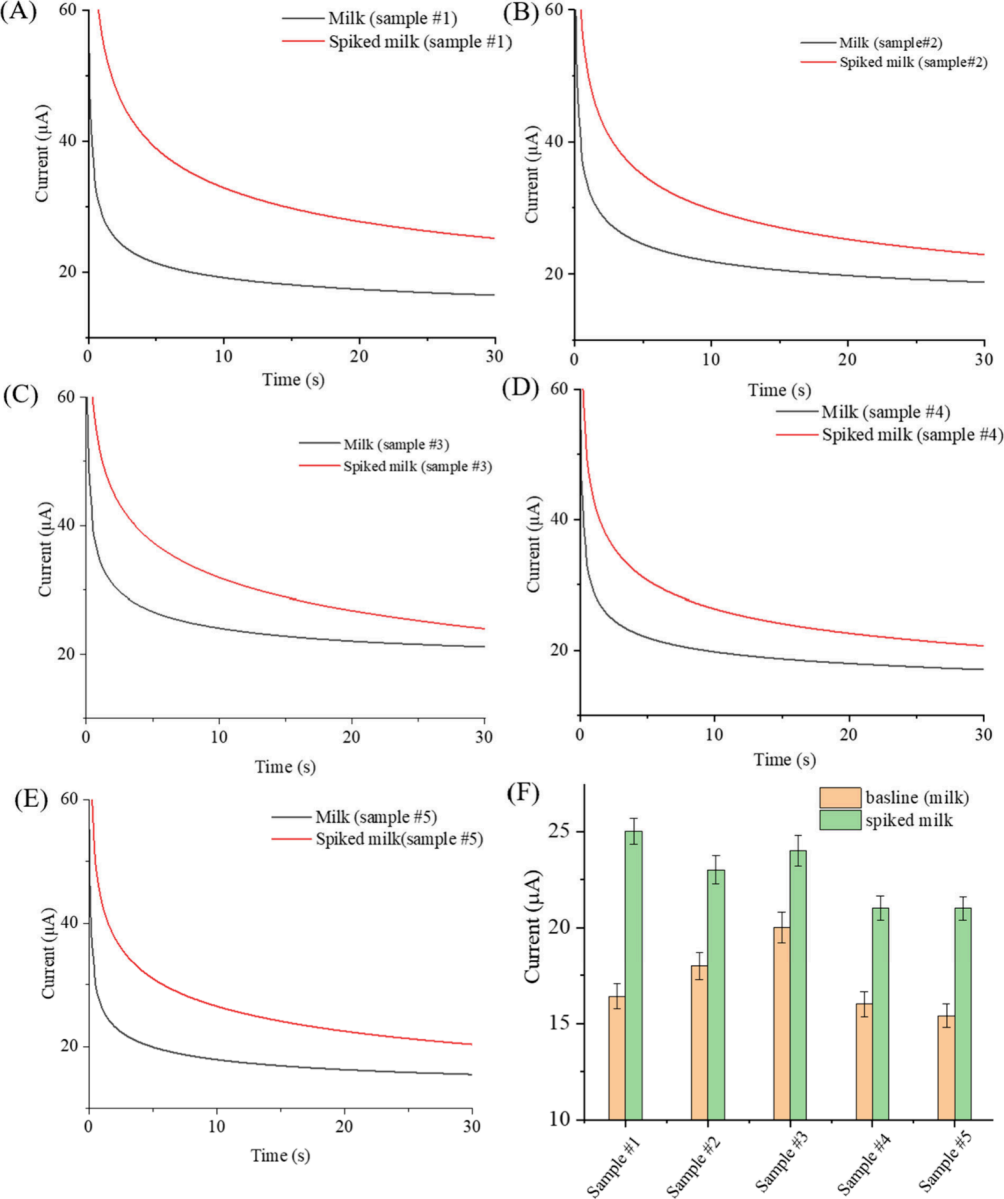
Real sample analysis. Chronoamperometric responses for real sample analysis (A–E) depict measurements, while (F) presents current overlays of the samples. This study utilized discarded milk samples from five lactating cows sourced from the Virginia Tech Kentland dairy complex, stored frozen until use. Each sample was spiked with βHB (1.50 mm) and mixed with a buffer solution of ferro/ferricyanide at a 1:1 ratio. Experiments were conducted both with and without βHB spiking for each sample. The sensor exhibited a relative standard deviation (RSD) of ±5.4% across all milk samples, indicating consistent performance. Variations in baseline current were observed due to differences inherent in milk samples. Sample #3 displayed higher current, possibly indicating the presence of βHB. Across all spiked βHB concentrations, the sensor maintained an RSD of ±3.9%, featuring its reliability and precision in quantifying βHB levels in milk samples.

As milk is a complex biofluid matrix,^60^ our Keto-sensor was tested with real milk samples to evaluate the sensor’s performance. Chronoamperometric responses of the Keto-sensor were obtained during the real sample analysis (Figure 6**)**. For this study, milk samples were obtained from five lactating cows at Virginia Tech’s Kentland dairy farm. These samples, stored frozen until use, were sourced as discarded samples. Each sample was spiked with βHB at a concentration of 1.50 mM and mixed with a buffer solution containing ferro/ferricyanide in a 1:1 ratio. The individual measurements conducted for each sample are shown in Figure 6A–E, while Figure 6F presents overlaid current traces from all samples. Both spiked and non-spiked samples were tested to validate the sensor’s performance. The sensor demonstrated a relative standard deviation (RSD) of ±5.4% across all milk samples, indicating consistent and reliable performance. Variations in baseline current were noted, reflecting inherent differences among milk samples. Notably, Sample #3 exhibited higher current levels, suggesting a potential presence of βHB. Across all spiked βHB concentrations, the sensor maintained an RSD of ±3.9%, indicating its precision in quantifying βHB levels in milk samples. Since these samples were obtained from nonketotic cows, glucose levels were not measured in the milk samples. Future efforts will focus on collecting samples from ketotic cows and measuring glucose levels to establish a correlation with βHB levels.

## 4 Conclusions

In summary, this Keto-sensor shows promising results as it can detect both clinical and SCK in the serum of dairy cows with a response time of less than a minute. Detecting βHB at such a low concentration (0.01 μm) will allow farmers to monitor the changes in their cows’ metabolism before any problems arise. Additionally, the fast response time is ideal for field use of this sensor. This Keto-sensor also shows promise in the lab setting displaying differences between samples spiked and not spiked with βHB. Additionally, testing continuous measurement over longer periods will allow for continuous ketone sensing. Selectivity testing should continue with more biological compounds to further prove the good selectivity of the ketosis sensor. The limitations to on-farm use of this device include the need for proper training of farmers in its use and interpretation of results. Precision agriculture management is the future, as the world needs to produce more food using less land and fewer animals for our growing population.^61^ Precise and accurate biosensors, like the one developed, will help support sustainable agricultural production across the globe.

## Data Availability

All relevant data that support the findings of this study are presented in the manuscript and Supporting Information. The source data are available from the corresponding author upon request.

## Supporting Information Available

Additional details on chemical reagents, calculations of limit of detection, and figures of additional experiments and device setup (PDF)
