# Supplementary material for: Functionalized Graphene-Based Biosensors for Early Detection of Subclinical Ketosis in Dairy Cows
Source: ACS Appl Mater Interfaces. 2024 Aug 22;16(39):51932–43. doi: 10.1021/acsami.4c07715 (PMC11450706; doi:10.1021/acsami.4c07715)
Supplement: Supplementary file 1 — am4c07715_si_001.pdf [file am4c07715_si_001.pdf]

Supporting Information

Functionalized graphene-based biosensors for early detection of subclinical ketosis in dairy cows

Shannon Chick,[1] Matin Ataei Kachouei,[1] Katharine Knowlton*[1], and Md. Azahar Ali*[1,2]
[1]*School of Animal Sciences, Virginia Tech, Blacksburg, VA 24061*
[2]*Biological Systems Engineering, Virginia Tech, Blacksburg, VA 24061*

Keywords: Precision livestock farming, Subclinical Ketosis, Biosensor, Graphene, Stabilized Enzyme

Corresponding Authors: Md. A. Ali, Email: azahar@vt.edu, phone: +1–540–231–5253
; K. Knowlton, Email: knowlton@vt.edu, phone: +1–540–231– 5287

Section 1: Chemical Reagents

βHBD, NADH, EDC, NHS, glycerol, and fetal bovine serum (FBS) were purchased from Sigma Aldrich, MO, USA. Highly dispersed graphene oxide solution of 0.5 mg/mL concentration was prepared in deionized (DI) water by adding 5 mg of graphene oxide nanosheets (ACS Material, CA, USA) to 10 mL of DI water. These 1-5 atomic layer graphene nanosheets were produced via thermal exfoliation followed by hydrogen reduction. The size of these nanosheets varied from 0.5 µm to 5 µm with conductivity of 500~700 S/m and BET surface area of 650~750 m^2/g. Phosphate buffer saline solution (PBS) was prepared by adding 6 grams of potassium phosphate monobasic (Fisher Chemical, MA) in 250 mL (0.2 M solution) of DI water and 7.1 grams of sodium phosphate dibasic anhydrous (Fisher Chemical, MA) added into 250 mL of DI water (0.2 M solution), then 19 mL of the monobasic solution was added to 81 mL of the dibasic solution to reach a pH of 7.4. Lastly 100 mL of DI water was added to create the PBS. The NHS stock solution was made by adding 28.7 mg NHS (Sigma Aldrich, MO, USA) to 5 mL PBS (0.05 M solution) while EDC stock solution was made by adding 0.177 mL EDC (Sigma Aldrich, MO, USA) to 4.82 mL PBS (0.2 M solution). The stock solution of βHBD was prepared by adding 1 mL PBS to 25-units of βHBD (Sigma Aldrich, MO, USA). NADH stock solution was made by adding 1 mL PBS to 25 mg NADH powder (Sigma Aldrich, MO, USA). DI water, ACS reagent grade, ASTM Type I (Lab Chem, PA) having a resistance of 18.2 MΩ was used to make buffer, sensing solution, and spiking tests A stock solution of 25 mM βHB was created by the addition of 25 mg of βHB (Sigma Aldrich, MO) to 20 mL of phosphate buffer saline (PBS) solution. To make a buffer solution for sensing experiments, 1800 mg (0.9%) of NaCl (Fisher Chemical, MA), 329.26 mg of potassium ferricyanide (III) (Sigma-Aldrich, MO), and 422.39 mg of potassium hexacyanoferrate (II) trihydrate (Thermo Fisher Scientific, MA) was added to the PBS and mixed until dissolved. This provides an equimolar concentration (5 M) of ferro/ferricyanide electrolyte probe to conduct our experiments.

Section 2: Calculation of limit-of-detection (LoD)

The LoD was calculated for the screen-printed, graphene, and ketosis sensor as reported by Armbruster. The limit-of-blank was calculated by **Equation (S1).**

(S1) LoB = Mean of signal (blank sample) + 1.645 × (Standard deviation of blank sample)

LoB for screen-printed sensor = 138.10 + 1.645 × 7.75 = 150.85

LoB for graphene sensor = 33.48 + 1.645 × 2.12 = 36.96

LoB for ketosis sensor = 50.64 + 1.645 × 7.61 = 63.15

Next, the limit-of-detection (LoD) of the sensors were calculated by **Equation (S2).**

(S2) LoD of the signal (Y_{LOD}) = LoB + 1.645 × (Standard deviation of target at low concentration)

Y_{LOD} for screen-printed sensor = 150.85 + 1.645 × 5.70 = 160.23

Y_{LOD} for graphene sensor = 36.96 + 1.645 × 2.10 = 40.40

Y_{LOD} for ketosis sensor = 63.15 + 1.645 × 3.87 = 69.51

Using the calibration curve of each sensor, the Y_{LOD} for each sensor was calculated using **Equation (S3).**

(S3) Y_{LOD} (Ω) = mX + c

m is the slope of the calibration curve and c is the intercept.

Y_{LOD} (Ω) for the screen-printed sensor = 48.8 × Log [X] +163.23

Y_{LOD} (Ω) for the graphene sensor = 26.6 × Log [X] + 47.4

Y_{LOD} (Ω) for the ketosis sensor = 6.52 × Log [X] + 93.01

From **Equations (S2)** and **(S3)**,

Log [X (nm)] for the screen-printed sensor = (160.23 – 163.23)/48.8 = -0.067

LOD = 857.02 nM

Log [X (nm)] for the graphene sensor = (40.40 – 47.4)/26.6 = -0.263

LOD = 545.56 nM

Log [X (nm)] for the ketosis sensor = (69.51 – 93.01)/ 6.52 = -3.604

LOD = 0.24 nM

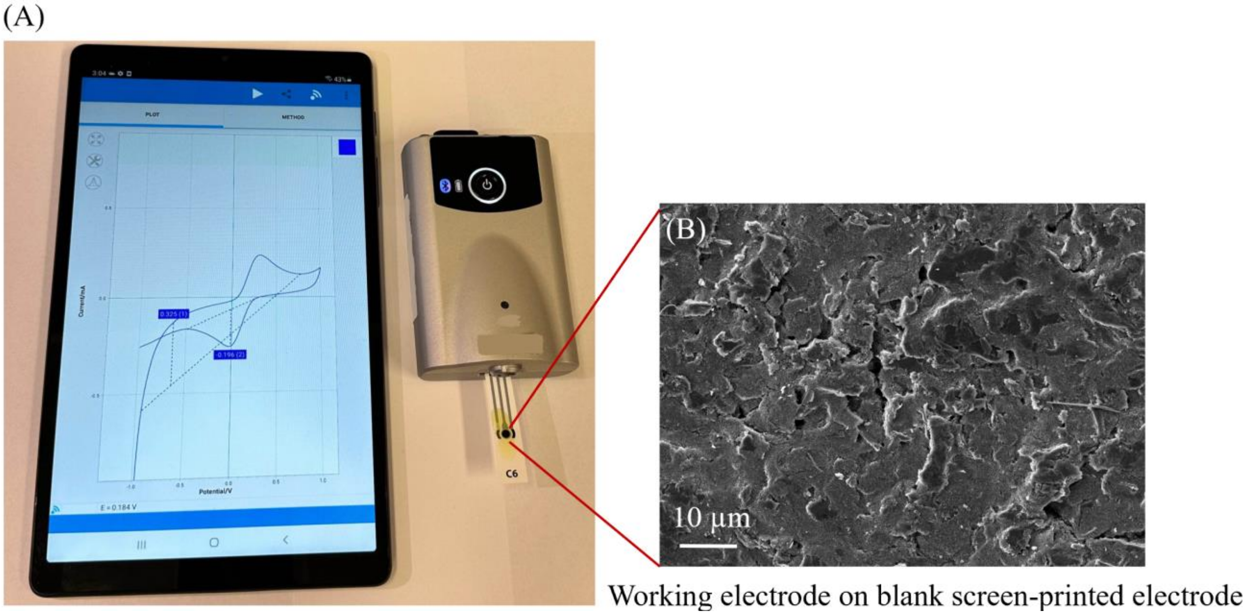

Figure S1: (A) Image of ketosis sensor and potentiostat connected via Bluetooth to a mobile tablet. (B) SEM image of the working electrode on the blank screen-printed sensor.

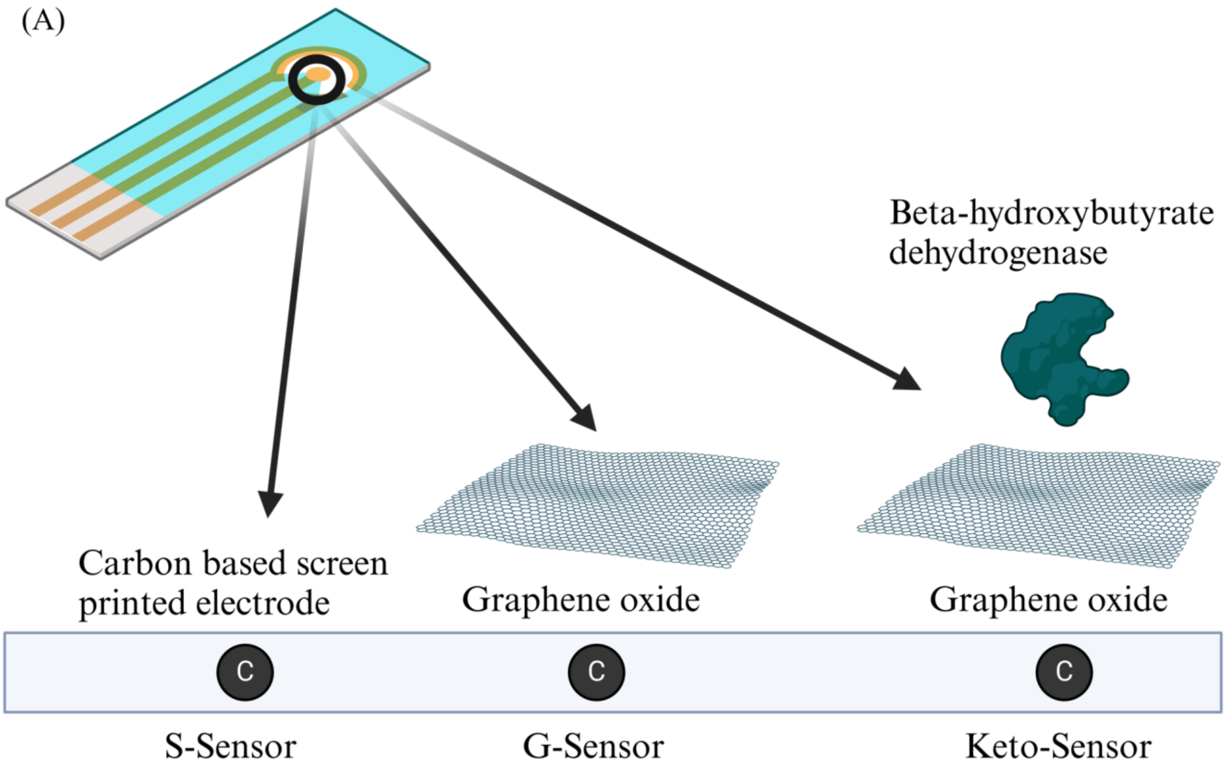

Figure S2: (A) Schematic representation of the WEs of the S-sensor, G-sensor, and Keto-sensor.

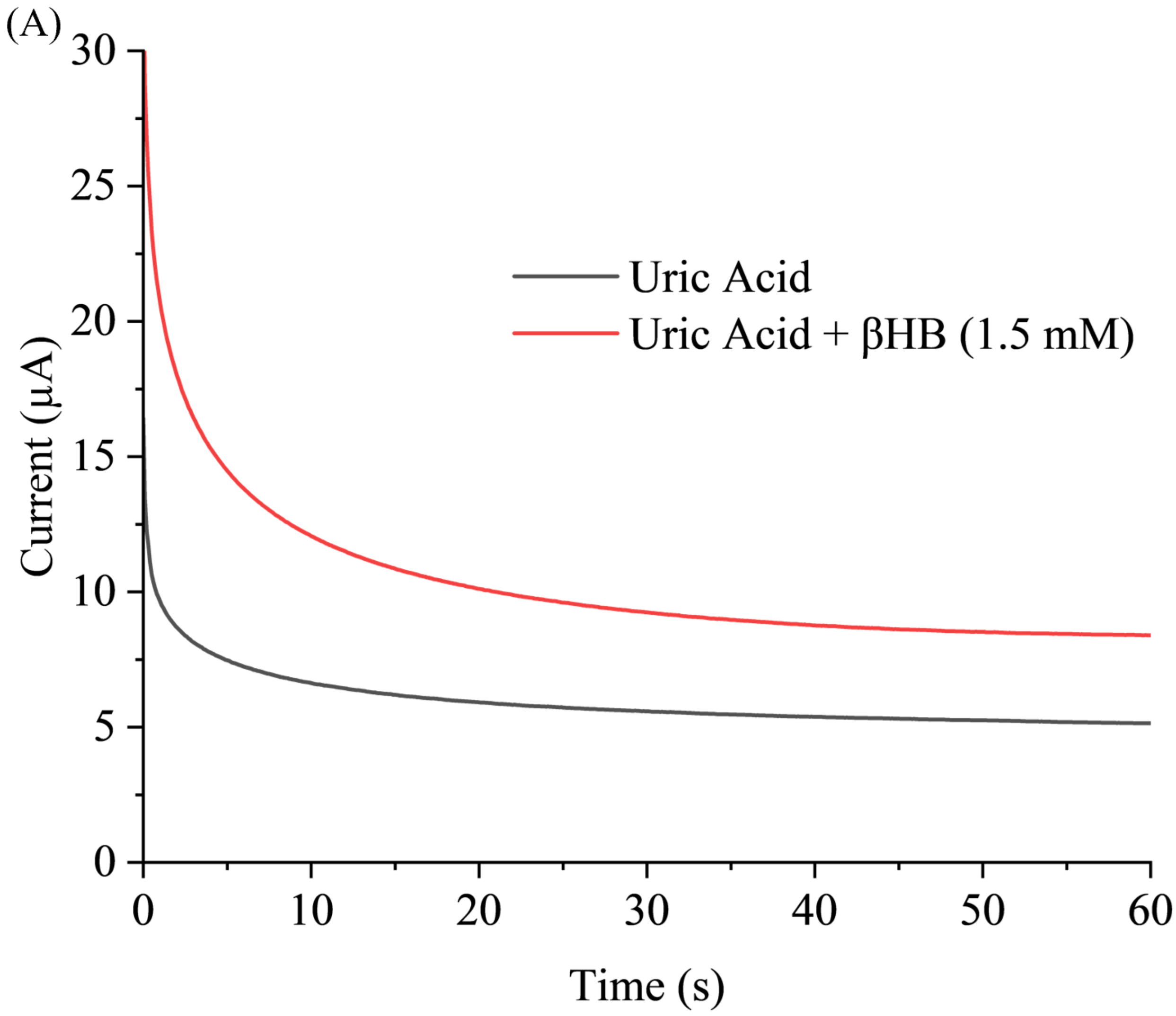

Figure S3: (A) CA plot from selectivity test using uric acid solutions of 13.00 mg/L concentration with and without 1.50 mM of βHB added. With the spiked uric acid solution, the current increased in comparison to the non-spiked uric acid solution.

Reference

(1) Armbruster, D. A.; Pry, T. Limit of Blank, Limit of Detection and Limit of Quantitation. *The clinical biochemist reviews* **2008**, *29* (Suppl 1), S49.
